# Oscillatory penetration of near-fields in plasmonic excitation at metal-dielectric interfaces

**DOI:** 10.1038/srep24400

**Published:** 2016-04-19

**Authors:** S. C. Lee, J. H. Kang, Q-H. Park, S. Krishna, S. R. J. Brueck

**Affiliations:** 1 Center for High Technology Materials and Department of Electrical and Computer Engineering, University of New Mexico, Albuquerque, NM 87106, USA; 2 Department of Physics, Korea University, Seoul 136–701, Korea

## Abstract

The electric field immediately below an illuminated metal-film that is perforated with a hole array on a dielectric consists of direct transmission and scattering of the incident light through the holes and evanescent near-field from plasmonic excitations. Depending on the size and shape of the hole apertures, it exhibits an oscillatory decay in the propagation direction. This unusual field penetration is explained by the interference between these contributions, and is experimentally confirmed through an aperture which is engineered with four arms stretched out from a simple circle to manipulate a specific plasmonic excitation available in the metal film. A numerical simulation quantitatively supports the experiment. This fundamental characteristic will impact plasmonics with the near-fields designed by aperture engineering for practical applications.

Since Ebbesen and coworkers first reported extraordinary transmission through a metal film perforated with an array of subwavelength holes, there have been substantial efforts to understand the interaction of visible- to millimeter- electromagnetic waves with such metal films, referred to in this work as metal photonic crystals (MPCs)^1^,^2^. It is well known that there are resonant contributions to the near-fields consisting of both propagating surface plasma waves (SPWs) bound to the metal/dielectric interface, which are primarily sensitive to the periodicity of the array, and localized plasma resonances (LPRs) associated with the details of the hole geometry. In combination, SPW and LPR are referred to as SR below.

For a square hole array patterned MPC on a semi-infinite dielectric, multiple SPW excitations are available at wavelengths given by^2^:
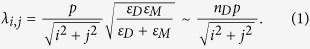


Here, ε_*D*_ and ε_*M*_ are the dielectric constants of the dielectric and metal, *n*_*D*_ = 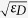, *p* is the spatial periodicity of the hole array in the MPC, and (*i*, *j*) = (±1, 0), (0, ±1) for the first-order SR (SR1), (±1, ±1) for the second-order SR (SR2), and so on for higher resonances. Most of the studies of the SRs have been focused on the physics of far-field phenomena which are now fairly well understood. On the other hand, the study of near-field effects has been much more limited. The near-field is very important for the complete understanding of plasmonic excitation in these structures. Also, nanoscale electronic and optoelectronic devices, such as photodetectors and solar cells, where the light-matter interaction involved in the characteristic performance occurs within the penetration depth of the SPW from the metal surface, are critically dependent on the details of the near-field^3^,^4^.

There have been many reports aimed at understanding the interaction between plasmonic excitations of an MPC and their impact on the reflected/transmitted/scattered light. The interference of SPWs and LPRs has been studied as it influences these far-field measurements^5^,^6^,^7^. Also, several articles have studied the interaction of the LPR evanescent field excited by a single aperture in a metal film with directly transmitted light through the film for metals with thicknesses less than the skin depth^8^,^9^,^10^. In contrast, this work examines how the SPW near-field penetrates into a dielectric from the MPC/dielectric interface under the variation of hole shape and dimension of an MPC that accompanies the interaction of SPWs with the incident light directly transmitting through individual holes. For this purpose, we investigate long wavelength infrared where the skin depth is very short compared with typical deposited metal thickness for plasmonic couplers. For such an MPC, the SPW field, bound to the interface, exhibits an evanescent, exponential decay into the dielectric with no transmission through the metal film. By increasing aperture size, we observe in the simulation that the near-field at shorter wavelengths, rather than monotonically decreasing from the interface, shows an oscillatory behavior. This unusual field penetration is interpreted as arising from interference between the SPW near-field and the transmission and scattering of incident light directly through individual apertures which result in far-field propagating transmitted and scattered plane waves beyond the MPC. This characteristic phenomenon, referred to as near-field interference (NFI), is of fundamental interest in the understanding of plasmonic interactions at a metal-dielectric interface and of engineering importance in optimizing the light absorption in the active regions of semiconductor devices.

Basically, direct transmission is related with aperture shape and size^11^,^12^,^13^. As the SR order increases, the aperture dimension scaled to the resonant wavelength is relatively larger as is evident from (1) and therefore direct transmission/scattering through the hole becomes greater. Engineering the aperture shape from a simple circle to a “Celtic cross” (CX) having four arms (width 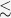
*p*/2) stretched out from the circle, we experimentally demonstrate NFI with a near-field design for a specific SPW that couples to an intersubband transition of a quantum dot infrared photodetector (QDIP). We present an electromagnetic simulation that explains this photoresponse with an oscillatory, non-monotonic decay of the SPW by the NFI enabling a manipulation of the near-fields coupled to the photodetector absorption region.

## Results

### Simulation of NFI

**Figure 1:**
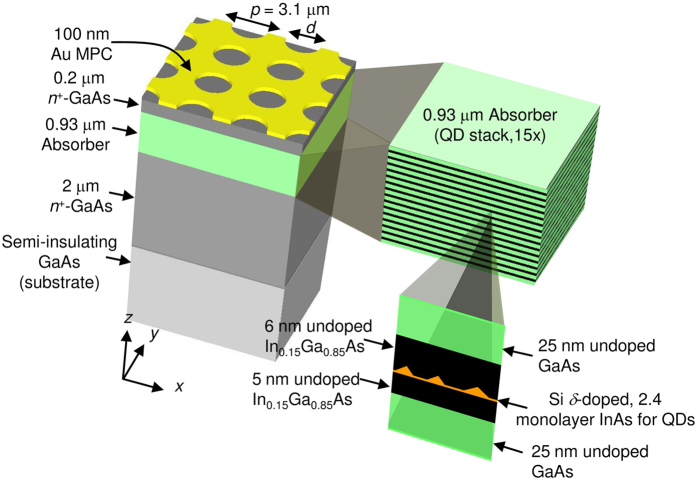
A schematic illustration of a device structure consisting of an MPC and a QDIP. The device structure on the left with a 3 × 3 circular hole MPC at the top is used for the simulation. The absorber on the right shows the detailed structure used in the experiment with a magnification of a single QD stack. In the simulation, these 15 QD stacks were regarded as a single layer with averaged material properties for convenience.

We consider a 100-nm thick gold MPC with a 2-dimensional (2D) square array of circular apertures of diameter *d*, and evaluate the near-fields by a finite-difference time-domain (FDTD) method. For comparison with experiment, the MPC simulation includes an InAs QDIP. Figure 1 is a schematic illustration of the device structure used in both the simulation and the experiment that is aligned to the *z*-axis. It consists of an MPC and a 930 nm-thick absorber (15 stacks of InAs QD layers) sandwiched by a 200 nm-thick and a 2 μm-thick *n*^+^-GaAs layer (for top and bottom ohmic contacts) on a semi-infinite undoped GaAs substrate (see Methods for detailed structure). In (1), *n*_*D*_ ~ 3.3 of the GaAs-based QDIP employed in this work. By setting *p* = 3.1 μm, the wavelengths of SR1 and SR2, *λ*_SR1_ (=*λ*_0,1_) and *λ*_SR2_ (=*λ*_1,1_), become ~10 μm and 7 μm from (1) respectively. At these wavelengths, the Au skin depth (<30 nm) is considerably smaller than the 100 nm film-thickness and direct transmission through the MPC is negligible^14^.

**Figure 2:**
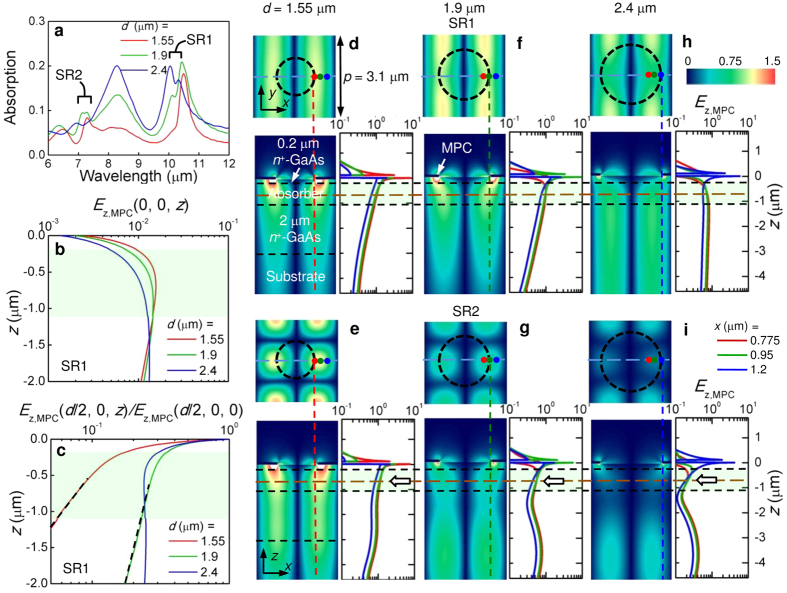
Simulation of NFI with circular aperture MPCs. (**a**) A plot of absorption vs wavelength of a 100 nm-thick Au MPC with a 2D square array of circular holes on the QDIP for *d* = 1.55, 1.9, and 2.4 μm. (**b**) A plot of *E*_*z,MPC*_ of SR1 vs *z* at the center of the circular aperture of three circles obtained from the side-view field map in (**d**), (**f**) and (**h**). (**c**) A plot of normalized *E*_*z,MPC*_ at *x* = *d*/2 vs *z* in each circle obtained from the depth profile in (**d**), (**f**) and (**h**). The dashed line on each curve follows the segment that was used for the calculation of *δ* in (2). 3D maps (left) and depth profiles (right) of |*E*_*z,MPC*_| at SR1 (top) and SR2 (bottom) with circular apertures of diameters, (**d**), (**e**) *d* = 1.55 μm (*p*/2); (**f**), (**g**) *d* = 1.9 μm; (**h**), (**i**) *d* = 2.4 μm. In each figure, the top row is a *xy*-plan view of the unit cell (3.1 × 3.1 μm^2^) at the middle of the absorber (along brown dashed lines at *z* = −0.565 μm in side view) and the bottom row is a *zx*-side view (along a light-blue dashed line in each plan view). The depth profile in each figure presents the variation along the selected dashed lines at *x* = 0.775, 0.95, and 1.2 μm (red, green, and blue dots in each plan view), corresponding to the aperture edges of three circles (*d*/2). A shaded region in each plot denotes the absorber. The incident light is polarized along the *x*-axis and *κ*_*A*_ = 0.03 was assumed.

SR excitation at an MPC/dielectric interface at normal incidence vanishes in the two extreme limits of an infinitesimally small aperture or an aperture as large as the pattern unit cell. Thus, SRs have the highest evanescent fields when the aperture size is comparable to *p*/2. In the simulation, therefore, three MPCs with circular apertures having diameters, *d* = 1.55 μm (=0.5*p*), 1.9 μm (~0.6*p*), and 2.4 μm (~0.8*p*), are examined. For convenience, these are referred to as small, medium and large circles. The details of the FDTD method used in this work have been reported elsewhere^15^,^16^,^17^. The material parameters used in the simulation are summarized in Methods. The only scaling parameter in the simulation was the imaginary part in the refractive index of the absorber, *κ*_*A*_, which shows a good agreement with the experiment at 0.03 (see Methods for discussion of *κ*_*A*_). Figure 2a shows the absorption spectra obtained from a calculation of 1-*R*-*T* with *κ*_*A*_ = 0.03 where *R* and *T* are the reflection and transmission of the full structure. This includes the absorption by both QD stack and the metal film. As indicated in Fig. 2a, there are two pronounced peaks for SR1 and SR2 around 10 μm and 7 μm, corresponding to *λ*_SR1_ and *λ*_SR2_, for each circle from (1), with an additional peak at 8.3 μm dominant at large *d*. This peak will be discussed later. A splitting of the SR1 peak is observed as *d* is increased.

Figure 2d–i present the 3-dimensional (3D) field map and depth profile of |*E*_*z,MPC*_(*x*, *y*, *z*)|, the magnitude of *z*-component of the electric field in the device, at *λ*_SR1_ (d, f, and h) and *λ*_SR2_ (e, g, and i) for the small, medium, and large circular aperture MPCs. Here, the light is normally incident toward an MPC from +*z* with a magnitude of unity polarized along the *x*-axis indicated in Fig. 2, and *z* = 0 corresponds to the MPC/QDIP interface. The top (bottom) panels show |*E*_*z,MPC*_| in a *xy* (*xz*) plane in the middle of the absorber (*z* = −0.565 μm) [at the center of the unit cell along *z* (−4.6 μm ≤ *z* ≤ 1.8 μm)] for each aperture. The figure focuses on the *z*-directed field because it is entirely the result of plasmonic excitation and as seen later is particularly important to the QDIP because of the polarization dependence of the QD absorption. In each side view, |*E*_*z,MPC*_| has its highest value at *z* ~ 0 and decays into the device. For better contrast below the MPC, the magnitude is truncated at 1.5. |*E*_*z,MPC*_| greater than this value is filled with white. The complete variation at the selected points of the red, green, and blue dots (*x* = 0.775, 0.95, and 1.2 μm), corresponding to the aperture edges of three circles (*d*/2), is presented in each depth profile.

The cross sections in Fig. 2 reveal two noticeable tendencies of *E*_*z*_ depending on *d*. For SR1 in Fig. 2(d,f,h), |*E*_*z,MPC*_| decays into the semiconductor at a rate that is more pronounced at smaller *d*. This tendency is confirmed by examining *E*_*z,MPC*_(0, 0, *z*) of SR1 along the center of each aperture over the range of *z* covering the absorber of the photodetector, as shown in Fig. 2b. The null of *E*_*z,MPC*_ (~0) at the center of the aperture reflects the standing wave field of counterpropagating SPWs, excited at normal incidence. Figure 2c is the magnification of the *E*_*z,MPC*_ in the depth profiles of SR1 normalized by *E*_*z,MPC*_(*d*/2, 0, 0) for individual circles. From Fig. 2b,c, |*E*_*z,MPC*_| of SR1 can be written as
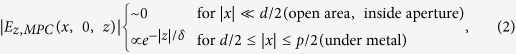
for *z* < 0 with penetration length, *δ*, = 0.79 ± 0.01 μm and 1.61 ± 0.05 μm for the small and medium circles at the aperture edge from the fitting indicated with dashed lines in Fig. 2c respectively. It should be noted that the decay rate of these fields in (2) is much faster than that estimated from a blanket metal film-semiconductor interface. As seen in Fig. 2c, the ^|^*E*_*z,MPC*_*|* for all three circles has an additional, extremely localized contribution just at the MPC/QDIP interface (steeper than the SPW evanescent decay into the semiconductor) at |*x*| ~ *d*/2 near *z* ~ 0 (~aperture edge) that is related to LPR. As expected, the *E*_*z,MPC*_ of SR1 that is weak at *x* ~ 0 inside the aperture along the *z*-axis and retains the expected exponential dependence below the metal area of each MPC is primarily due to the evanescent SPW decay.

For SR2, on the other hand, there is a clear oscillation in ^|^*E*_*z,MPC*_| that becomes more pronounced at larger *d*. The characteristic decay of the SPW field, bound to the interface, observed in SR1 is no longer maintained for the shorter λ_SR2_. The depth profile for all three apertures in Fig. 2(e,g,i) clearly reveals this behavior with a first maximum appearing for *z* ~ −3.0 to −3.5 μm as a function of *d*. As indicated by the white arrow at the middle of the QD absorber in each SR2 depth profile, this results in a reduced field and therefore a weaker photoresponse at *λ*_SR2_ in plasmonic coupling. The large circle even shows a hint of this behavior at SR1 in the normalized depth profile of Fig. 2c. This means the oscillatory penetration is a fundamental characteristic of the SPWs excited at an MPC/dielectric interface.

A possible explanation is destructive interference of the SR near-fields with light directly transmitted through and scattered by each aperture into propagating modes in the semiconductor. This interference leads to the variation in *E*_*z,MPC*_ in the QDIP. In contrast to the results of refs 8, 9, 10, in this case the interference is between the SPW and the fields scattered from individual apertures that collectively result in the far-field transmission. Thinking of a single circular aperture in a metal film as a dielectric loaded circular waveguide, the maximum cut-off wavelength for direct transmission, *λ*_*c*_, can be approximated as
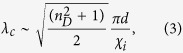
where *χ*_*i*_ = 1.841 is the first root of the first-order Bessel function^18^,^19^. At a given *d*, the direct transmission becomes more significant for incident light wavelengths shorter than *λ*_*c*_. Then, *λ*_*c*_ ~ 6.5, 8.3, 10 μm for *d* = 1.55, 1.9, and 2.4 μm, respectively, with *n*_*D*_ ~ 3.3 from (3). For the large circle, both SR1 and particularly SR2 are affected by direct transmission since *λ*_*c*_ ~10 μm ~ *λ*_SR1_ > *λ*_SR2_. For the medium circle, *λ*_SR1_ *>* *λ*_*c*_ *~*8.3 μm > *λ*_SR2_, SR1 is relatively unchanged by NFI while SR2 is clearly affected by it, as seen in Fig. 2g. For the small aperture, *λ*_SR1_ > *λ*_SR2_ ∼ *λ*_*c*_ ~ 6.5 μm, and there is negligible direct transmission for either resonance but, as seen in Fig. 2e, SR2 shows a weak oscillatory variation because *λ*_SR2_ is close to its *λ*_*c*_ ~ 6.5 μm. Therefore, the enhanced direct transmission with increasing *d*, or decreasing SR wavelength at a given *d*, can explain the oscillating *E*_*z,MPC*_ in Fig. 2. Such variation of *E*_*z,MPC*_ is also clear evidence for the presence of a near-field that decays away from the metal/dielectric interface.

### Experiment of NFI by aperture engineering

**Figure 3:**
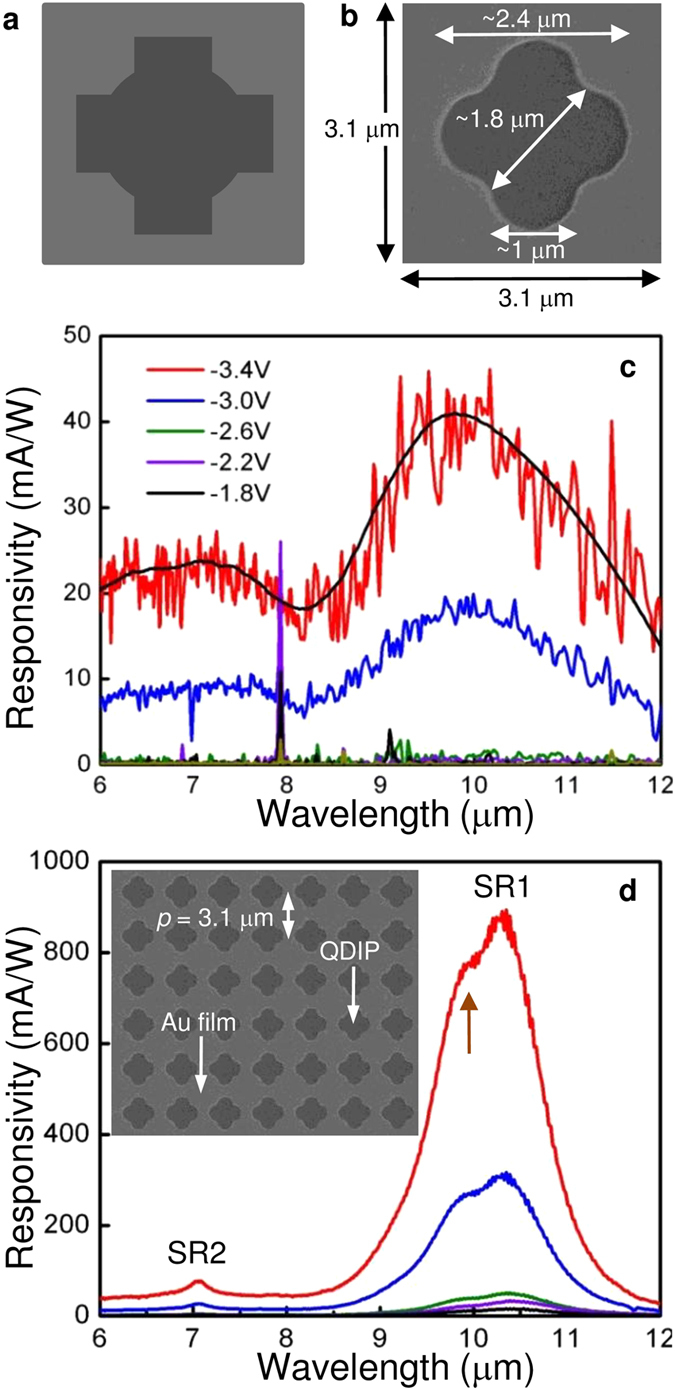
Experiment demonstrating NFI with aperture engineering. (**a**) An example of a Celtic cross (CX) aperture designed for the suppression of the SR2- while retaining the SR1-resonance. (**b**) A SEM image of a hole fabricated in this work that mimics (**a**). Plots of experimental responsivity vs. wavelength of (**c**) the reference device and (**d**) the CX device. Negative bias means grounding the top contact in Fig. 1a. In (**c**), the black line on the curve for bias of −3.4 V was used in the simulation to scale *κ*_*A*_. In (**d**), a brown arrow indicates a shoulder at ~9.7 μm. The color code in (**c**) is identically applied to (**d**). Inset in (**d**) A SEM image of the 2D array in the MPC with the CX aperture in (**b**) that was integrated atop the QDIP semiconductor structure.

In Fig. 1, the incident light is resonantly coupled to an SPW if its wavelength satisfies (1) for the plasmonic excitation at the MPC/QDIP interface that result in evanescent near-fields interacting with the absorber underneath the MPC and therefore directly revealing NFI from the variation of the photoresponse of the detector. In Fig. 2, *E*_*z,MPC*_ varies with *d* and SR orders. Such strength variation can be examined experimentally with the QDIP in Fig. 1 which has two-color response near *λ*_SR1_ and *λ*_SR2_. Based on the variation in Fig. 2, retaining an enhanced SR1 at *λ*_SR1_ while suppressing the response at SR2 at *λ*_SR2_ by NFI is available with a single MPC by aperture engineering. Figure 3a shows a basic aperture shape designed for this purpose, a circular hole with four arms stretched out along the pattern symmetry directions. This is similar to the union of a circle and a cross, referred to as a Celtic cross (CX). Figure 3b is a scanning electron microscope (SEM) image of the aperture fabricated for this work that mimics Fig. 3a. It has four ~1 μm-wide arms and a diagonal opening ~1.8 μm, longer than *p*/2. Its largest opening gap is ~2.4 μm as indicated. Its shape and overall pattern have four-fold symmetry, so the response is independent of the polarization of the incident light. It retains the strength of SR1 at the longest wavelength with the arms but allows direct transmission/scattering through individual apertures for SR2 and higher-order SRs with the extended aperture provided by these arms. As a result of lithographic limitations, most of the right-angle corners of Fig. 3a were not replicated in Fig. 3b but the overall shape retains the characteristics expected from the hole in Fig. 3a, as confirmed experimentally.

Fig. 3c,d show the photoresponse spectra from the two QDIPs with variation of the bias for the reference device, and for the 100 nm-thick Au film CX MPC-integrated device (CX device) respectively, measured at 10 K in normal incidence (see Methods for the QDIP fabrication and characterization). The inset of Fig. 3d is an SEM image of the MPC with an array of the CX apertures in Fig. 3b that is taken from the CX device. In Fig. 3c, the reference device has a two-color response with broad peaks centered at 10.0 and 7.2 μm, close to the wavelengths of SR1 and SR2 in Fig. 2a. As reported previously, these peaks are the result of transitions from the QD ground state to the first excited quantum well and to the continuum states^3^,^20^. The black line following the response curve at −3.4 V of Fig. 3c is taken as the spectral dependence of the absorption for κ_Α_ (see Methods for details).

The CX device of Fig. 3d has a strong peak at *λ*_SR1_ = 10.3 μm with only a very weak peak at *λ*_SR2_ = 7.1 μm. In Fig. 3c,d, the peak responsivity at −3.4 V where the detectivity of both devices has the maximum value is increased from 41 mAW^−1^ for the reference device to 873 mAW^−1^ in the CX device. The enhancement in responsivity is ~21×, consistent with previous work^3^,^21^. In Fig. 3d, however, SR2 has very low intensity compared with SR1. Then, the enhancement is exclusively by the coupling to SR1. This is expected from the aperture design and is very different from previous reports on a similar QDIP with a circular hole MPC where the SR1 and SR2 responses were comparable. Also, as indicated by an arrow in Fig. 3d, a shoulder is evident at ~9.7 μm on the strong peak of SR1.

### Comparison of experiment with simulation

**Figure 4:**
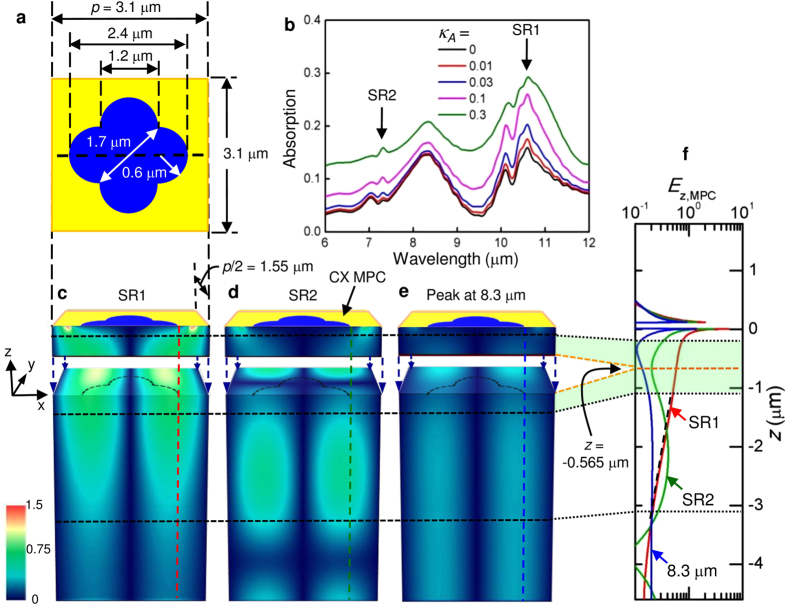
Simulation of NFI with a CX aperture MPC. (**a**) The hole shape used in the FDTD simulation that is similar to Fig. 3b. (**b**) A plot of absorption vs. wavelength of CX MPC with the aperture in (**a**) from the simulation along the variation of *κ*_*A*_ from 0 to 0.3. The brown arrow indicates the splitting in SR1. 3D maps of |*E*_*z,MPC*_| at (**c**) SR1, (**d**) SR2, and (**e**) 8.3 μm of an MPC with CX of (**a**) in *xy*- (*z* = −0.565 μm) and *zx* plane (−4.6 μm ≤ *z* ≤ 0) for *κ*_*A*_ = 0.03. The side-view is along the black dashed line in (**a**). The dashed shape on the *xy* plane is the projection of the CX at the top. (**f**) Depth profile of |*E*_*z,MPC*_| vs *z* at the edge of the CX aperture indicated with dashed color lines in (**c**–**e**). The dotted black lines across the side views are the layer interfaces shown in Fig. 2d, matching (**c**–**f**) in *z*. The dashed line on the red curve in (**f**) is the fitting segment used for *δ* in (2). A shaded region in (**f**) indicates the absorber. The orange dashed lines follow *z* = −0.565 μm in the middle of the absorber where the splitting of the *zx*-side view occurs to reveal the *xy*-plan view at the given *z* in the field map. The incident light is polarized along the *x*-axis.

The structure in Fig. 4a, imitating the CX shape of Fig. 3b, was used for FDTD simulation. This aperture has the same opening area as the medium circle with the same maximum lateral dimension as the large circle. Figure 4b shows the simulated total absorption spectra with *κ*_*A*_ scaled from 0 to 0.3 at 10 μm. As indicated by arrows in the figure, SR1 and SR2 are around 10.6 μm (=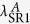) and 7.3 μm (=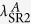) for *κ*_*A*_ = 0.03 which are only slightly different from *λ*_SR1_ and *λ*_SR2_ experimentally observed for the CX device in Fig. 3d. The material parameters assumed in the simulation could be a reason for this minor difference. The absorption of SR1 at *λ*_SR1_ increases with *κ*_*A*_ as expected. SR1 shows a splitting similar to that noticed experimentally in Fig. 3d. Another peak at ~8.3 μm appears in Fig. 4b as was observed in connection with Fig. 2a. This peak does not contribute to the photoresponse, as discussed later.

Figure 4c–f present the 3D field map and depth profile of ^|^*E*_*z,MPC*_| of the CX device for *κ*_*A*_ = 0.03 with incident light polarized along the *x*-axis as in Fig. 2. Depth profile follows the vertical color lines at the edge of the CX aperture in the field map. It should be noted that the field pattern of the polarization along the plane of x = y is different from that of y = 0 in Fig. 4c–e because of CX symmetry. This polarization can be decomposed into the components parallel to the planes of *x* = 0 and *y* = 0 (*yz* and *zx* planes) which are identical to each other under the given symmetry. Therefore, the field pattern along the plane of *x* = *y* can be conjectured from these figures. In Fig. 4c,f, SR1 of CX has a strong |*E*_*z,MPC*_| pattern across the gap for *x*-axis-polarized incident light, as expected. Furthermore, it fully covers the absorber with *δ* = 3.7 ± 0.1 μm from (2) before decaying into the substrate. This penetration length and others measured at the small and medium circles earlier are much less than the evanescent field skin depth ~10 μm (~*λ*_SR1_), estimated from an unpatterned gold film atop the QDIP^3^. This is another important result of this work. For improved SPW coupling, the actual skin depth [or *δ* in (2)] must be considered and the absorber should be within it. The location of the absorber is a critical factor in designing QDIPs and MPCs for optimal plasmonic coupling. Evidently, the *δ* of CX is one of the characteristics of SPWs favorable for the coupling of the QD stack to SR1. In 3D |*E*_*z,MPC*_| pattern, therefore, the CX device resembles the medium circle of Fig. 2f for SR1, and the presence of the arms and their width (or narrow gap) are important in retaining its resonance characteristics.

SR2 is totally different from SR1 in CX. At SR2 of Fig. 4d,f, the |*E*_*z,MPC*_| is rather close to that of the large circle in both plan view and depth profile. In these figures and along the green line in Fig. 4d, the depth dependence clearly exhibits an oscillatory behavior similar to the medium and large circles with a lower |*E*_*z,MPC*_| and therefore a reduced overlap with the absorber. These imply a weak SPW coupling to the QD stack to SR2 resulting from enhanced NFI by CX apertures. Consequently, as seen in Fig. 4a, both the arms with 1.2 μm-wide base and the center opening with 1.7 μm in diagonal dimension extended with these arms are optically large enough for SR2 to encourage the direct transmission/scattering at the shorter *λ*_SR2_. The increased lateral dimension of CX aperture is an important factor for the suppression of SR2.

The dramatic (~21×) plasmonic enhancement in QDIPs has been explained by the intersubband absorption depending on the polarization of the incident light^3^. The enhancement is approximately proportional to 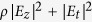, where *E*_*z*_ and *E*_*t*_ correspond to the electric field components along the *z*-axis and the *xy* plane in the absorber of Fig. 4 and *ρ* ~ 8 is a multiplier representing the enhancement for the interaction of a QD with *E*_*z*_ as a result of the dominant QD in-plane topology (e.g. transverse dimensions ≫ height)^22^. Previously, *ρ* was conservatively assumed to ~5 relying on the published data^3^,^23^,^24^, but is set to ~8 based on a recent measurement in ref. 22.

For normal incidence illumination, *E*_*z*_ ≡ 0 in the reference device of Fig. 3c and the photoresponse is only due to 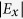
_2_. On the other hand, *E*_*z*_ ≠ 0 in the CX device in Fig. 3d by SR near-field and a large enhancement becomes available with *ρ*. In the CX device, the spectral enhancement by plasmonic excitation in the simulation, Γ_*s*_, with the light polarized in *x*-axis (*E*_*t*_ = *E*_*x*_) is therefore written as:
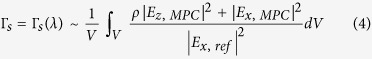
where the integration is over the volume of the absorber, *V*. Here, |*E*_*x,ref*_ | and |*E*_*x,MPC*_| are the transverse electric fields in the reference and CX devices respectively which are relatively similar, |*E*_*z,ref*_ | = 0 for normal incidence, and *ρ* is assumed constant across *V*.

**Figure 5:**
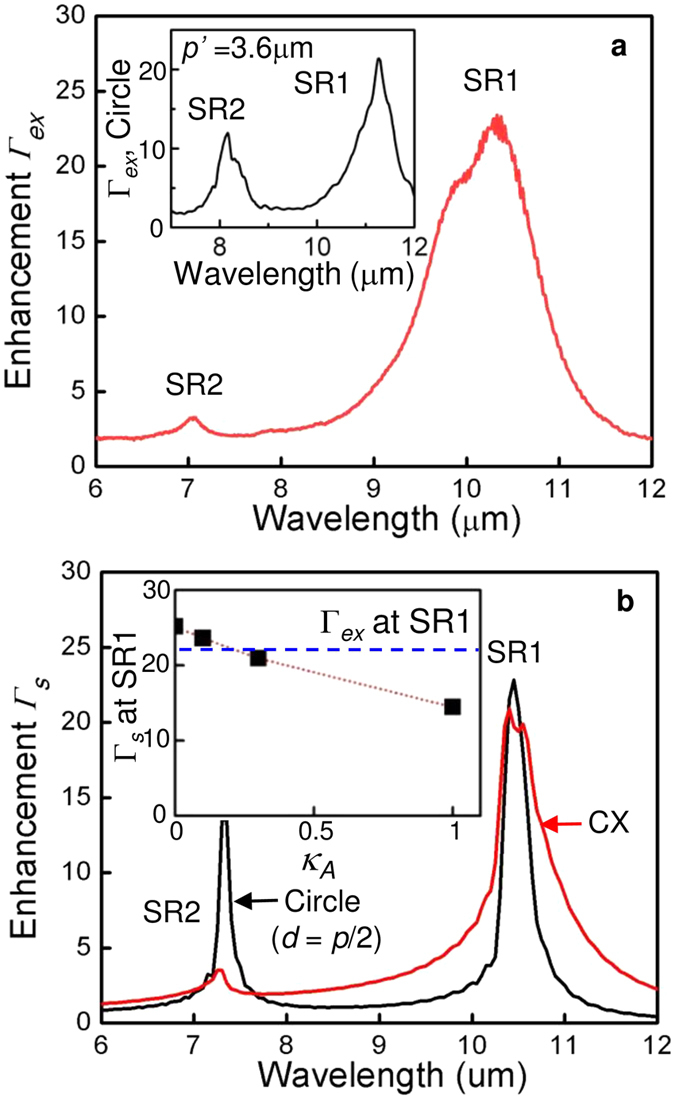
Comparison of experiment with simulation. (**a**) A plot of Γ_*ex*_ vs wavelength. Inset: A plot of Γ_*ex*_ vs wavelength from a similar QDIP with an MPC of a *p’* = 3.6 μm circular hole array (hole diameter ~ 1.7 μm ~*p*′/2) at the same bias polarity reported in ref. 3. (**b**) A plot of Γ_*s*_ vs wavelength with *κ*_*A*_ = 0.03. Inset: A plot of Γ_*s*_ at SR1 vs *κ*_*A*_ from 0 to 1. The dotted line is a guide for the eye. The dashed line corresponds to Γ_*ex*_.

Figure 5 presents plots of (a) Γ_*ex*_ and (b) Γ_*s*_ at *κ*_*A*_ = 0.03 versus *λ*. Here, experimental enhancement, Γ_*ex*_, is obtained from Fig. 3c,d through normalizing the spectrum of the CX device by that of the reference device at −3.4V and Γ_*ex*_ ~23 is the highest at 10.3 μm, identical to *λ*_SR1_. The inset in Fig. 5b shows the dependence of Γ_*s*_ at SR1 on *κ*_*A*_ from (4). When *κ*_*A*_ = 0, Γ_*s*_ is 26 at 10.4 μm, slightly offset from 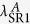.This result, however, merely refers to the integrated field strength across the absorber in (4) as there is no actual absorption for *κ*_*A*_ = 0. For *κ*_*A*_ > 0, the field decays more rapidly with *z* as a result of the increased absorption as seen in Fig. 4b; Γ_*s*_ at SR1 is reduced to 15 for *κ*_*A*_ = 0.1. Among the results in the inset of Fig. 5b, as mentioned earlier, Γ_*ex*_ is in the best agreement with Γ_*s*_ = 22.1 for *κ*_*A*_ ~ 0.03. A significant difference is the broadening in Γ_*ex*_ which is approximately twice that of Γ_*s*_. This could be due to the difference of the actual hole shape from the simulated one and to fabrication inhomogeneities.

The SR2 suppression ratio in the photo-enhancement by SPW coupling, *γ*, can be defined as the ratio of Γ at SR1 to that at SR2. Then, *γ*_*ex*_ for the experiment ~7.4 from Fig. 5a and is close to *γ*_*s*_ = 6.0 for the simulation from Fig. 5b for CX. The black curve in Fig. 5b is Γ_*s*_ obtained from the simulation with the small circle (*d* = 1.55 μm = *p*/2) in Fig. 2d for *κ*_*A*_ = 0.03. The numerical calculation for this aperture shows peak enhancement by SPW coupling for both SR1and SR2 in contrast to the CX which has an exclusively large enhancement at SR1 in agreement with the experimental result. For the circular aperture in Fig. 5b, *γ*_*s*_ = 1.7. This is almost the same as *γ*_*ex*_ ~ 1.8, obtained from a previously reported QDIP in the inset of Fig. 5a, which has a similar device epitaxial structure and an identically fabricated MPC except for a scaling of both the circular aperture diameter and the array pitch (hole diameter ~1.7 μm ~*p’*/2 with *p’* = 3.6 μm)^3^. Both *γ*_*ex*_ (~1.8 → 7.4) and *γ*_*s*_ (1.7 → 6.0) show ~4× improvement in SR2 suppression with similar Γ‘s (~22–24) at SR1 by changing the aperture shape from a simple circle to a CX. It clearly confirms NFI by aperture engineering. This is sufficient for single color detection by plasmonic excitation that is ultimately of practical interest for many applications such as infrared retina^25^. Although the simulation assumes an infinitely thick substrate and does not account for further scattering of this light back into the absorber, *i.e.* multiple pass effects, it quantitatively supports the experiment and provide strong evidence for NFI.

In Fig. 4c, *E*_*z,MPC*_ along the wide gap (2.4 μm in Fig. 4a) across the center is concentrated at the edge of the metal. The wide gap requires a higher energy in collecting charges for a dipole across the edges over the longer distance and induces a short-wavelength, weak SPW^13^. Then, two different SPWs should be available in a CX; a strong one across the narrow gap and another weaker one across the wide gap, as seen in the plan view of Fig. 4c. Figs 2a,3d,4b, and even 5b (CX) consistently show the peak splitting in SR1. In MPCs, there is a splitting due to symmetric SPWs propagating in opposite directions for normal incidence which generate standing waves and as a result a stopband in Bragg scattering^26^,^27^. This is available in the CX MPC and can exhibit an additional larger splitting associate with the more complex shape.

Finally, Fig. 4e shows the equivalent field map for the absorption peak at 8.3 μm, observed in Fig. 2a, for the CX of Fig. 4b that is not associated with any SRs in Fig. 5a,b for both apertures. Aside from some field suppression very near the MPC, the field strength is constant, indicating direct transmission/scattering, significantly weaker than the SPW fields. In Fig. 4b, the relative intensity decreases with increasing *κ*_*A*_, suggesting this absorption obtained from the calculation of 1-*T*-*R* seems to be related to the metal rather than the QDIP and does not contribute to the photoresponse.

## Discussion

In CX, the four arms are more critical to SR1 available at longer *λ*_SR1_ and the extended lateral dimension is more pertinent to SR2 occurring at shorter *λ*_SR2_. In a shape transformation from a *d* = 1.9 μm circle to CX without area change, all resonances except for SR1 are dominated by the direct transmission. Enhanced direct transmission/scattering means lowered SPW evanescent field strength for a given incident intensity. The coupling of the QDIP to SR2 and higher order SRs are thus suppressed. Alternatively, a transformation from a *d* = 2.4 μm circle to CX with keeping the largest gap equal to the diameter results in SR1 strengthened by the suppression of the direct transmission while leaving weak SR2 and higher order SRs unaffected. Both interpretations emphasize the significance of NFI by aperture engineering.

In summary, the oscillatory penetration of the near-field of the SPWs excited at an MPC/dielectric interface has been investigated. Based on the cutoff of cylindrical waveguide modes, such oscillatory behavior observed at higher SRs is interpreted by the interference of plasmonic excitation with direct transmission/scattering of incident light through individual holes. This has been experimentally confirmed with a metal film perforated with an array of CX apertures that specifically suppresses SR2 while maintaining SR1 in a single MPC. For CX, and similarly for circular apertures, the competition between far-field transmission and SPWs depends on the details of the aperture geometry and leads to a spatial reduction or an enhancement of the field intensity of SPWs. Particularly over the stretched arms having a width comparable to *p*/2 in CX, SR2 becomes weak by NFI enhanced with direct transmission. Both experiment and numerical calculation consistently show ~4× improvement in SR2 suppression with CX, sufficient to verify NFI. Hole shape engineering for NFI manipulation is available for further applications such as polarization-sensitive enhancement of individual SR modes by adjusting the width and length of the gaps. The introduction of resonant structures in the individual apertures can lead to phase changes in the various fields^7^ and hence to constructive as well as destructive interference, enhancing detector performance. Conclusively, this work clearly demonstrates fundamental and practical significance of the near-field of plasmonic excitations at metal-dielectric interfaces.

## Methods

### Simulation parameters

For undoped GaAs, the literature real part of the low-temperature refractive index (over the 7- to 12-μm wavelength range with imaginary part ~ 0) was used in the simulation^28^. For the refractive index of *n*^+^-GaAs (Si doping concentration ~ 2 × 10^18^ cm^−3^), the real part was adjusted for the dispersion of the bulk plasma contribution and the imaginary part (absorption) was set to *b*λ^4^ with *b* ~ 1.2 ± 0.1 × 10^−6^ μm^−4^ and wavelength, *λ*, in μm to account for absorption by free carriers^29^,^30^,^31^. Because the average amount of indium in the absorber is not significant (<4%), the *n*_*D*_ of undoped GaAs was used to characterize the absorber^32^. A Drude model was used for the refractive index of the Au film^32^.

The absorption by the contact layers and the semi-infinite undoped GaAs is negligible at the range of interest ~7–10 μm. The key unknown parameter required in the FDTD simulation is the imaginary part in the refractive index of the absorber, *κ*_*A*_, which impacts SR in both the propagation length along the interface and the field penetration depth into QDIP. Since the photodetection occurs through intersubband transitions, it was assumed that *κ*_*A*_(*λ*) = *κ*_0_*κ*(*λ*) where κ_0_ is a scaling factor and *κ*(*λ*) is assumed proportional to the spectral response of the QDIP in Fig. 3c. Then, only fitting parameter in the simulation is a scaling factor *κ*_0_. We set *κ* = 1 at *λ* = 10.0 μm on the black curve of Fig. 3c where the highest photoresponse occurs at the given bias and varies *κ*_0_ from 0 to 0.3, which is equivalent to the range of *κ*_*A*_. As seen in the inset of Fig. 5b, the simulation is close to the experiment for *κ*_*A*_ = 0.03 among the values in it.

### QDIP fabrication and characterization

The QDIP shown in Fig. 1(a) was grown by molecular beam epitaxy. The magnification at the right in Fig. 1 reveals the details of each QD stack consisting of a QD layer buried in an 11 nm-thick In_0.15_Ga_0.85_As quantum well layer along with a 50 nm-thick undoped GaAs spacer. For the QDs, 2.4 monolayers of InAs were deposited 0.5 nm below the center of the In_0.15_Ga_0.85_As layer. The QDs were Si*-δ*-doped at a nominal density of ~ 3 × 10^10^ cm^−2^. Two 410 × 410 μm^2^ mesa-type QDIPs were fabricated: one for the integration of an CX-aperture MPC (CX device); and the other for reference (with no MPC). Each was individually mounted in an isolated die so that it retains only a single 300-μm-diameter aperture for the incident light to avoid any issues with scattered light from adjacent device areas. For CX device, this aperture was covered by a 100 nm-thick Au film CX MPC while the reference device had a 300 μm diameter open aperture. Standard photolithography was employed for the MPC fabrication. Photoresponse was measured with Nicolet 6700 Fourier Transform Infrared Spectrometer, Stanford Research Systems FTT 770 network analyzer, and a black body source set to 800 K. Measurement temperature was set to ~10 K to reduce thermal noise.

## Additional Information

**How to cite this article**: Lee, S. C. *et al*. Oscillatory penetration of near-fields in plasmonic excitation at metal-dielectric interfaces. *Sci. Rep.* **6**, 24400; doi: 10.1038/srep24400 (2016).
